# Supplementary material for: End Sequence Analysis Toolkit (ESAT) expands the extractable information from single-cell RNA-seq data
Source: Genome Res. 2016 Oct;26(10):1397–410. doi: 10.1101/gr.207902.116 (PMC5052061; doi:10.1101/gr.207902.116)
Supplement: Supplemental Material [file supp_gr.207902.116_Supp_Materials.tar.gz › ESAT-esat_v0.1/doc/html/dir_f41cef3a2a07b14045e1ab3c3c685b01.html]

ESAT: java/broad/pda/geneexpression/agilent Directory Reference


ESAT


- Main Page
- Related Pages
- Packages
- Classes
- Files

All Classes Namespaces Files Functions Variables Enumerator Pages

- java
- broad
- pda
- geneexpression
- agilent

agilent Directory Reference

Directory dependency graph for agilent:

|  |  |
| --- | --- |
| Directories | |
| directory | analysis |
|  | |

|  |  |
| --- | --- |
| Files | |
| file | AgilentArrayStats.java |
|  | |
| file | AgilentProbe.java |
|  | |
| file | AgilentToGCT.java |
|  | |
| file | AgilentUtils.java |
|  | |
| file | ClassifyAgilentLincProbes.java |
|  | |
| file | CollapseProbesByMinPValue.java |
|  | |
| file | CorrelationAgilentNanostring.java |
|  | |
| file | CorrelationBetweenReplicates.java |
|  | |
| file | ExtractSamplesAndLabel.java |
|  | |
| file | FilterMatrixByMinCutoff.java |
|  | |
| file | FindProbesForGeneSet.java |
|  | |
| file | MakeCLSFiles.java |
|  | |
| file | MakeCLSFromDescription.java |
|  | |
| file | QCAgilentResults.java |
|  | |


---

Generated on Fri Aug 15 2014 15:11:15 for ESAT by  

 1.8.7
